# Physical Resilience as a Determinant of Healthspan and Lifespan in Mice

**DOI:** 10.1093/geroni/igaa057.3031

**Published:** 2020-12-16

**Authors:** Nathan LeBrasseur

**Affiliations:** Mayo Clinic, Rochester, Minnesota, United States

## Abstract

Dynamic measures of physical resilience—the ability to resist and recover from a challenge—may be informative of biological age far prior to overt manifestations such as age-related diseases and geriatric syndromes (i.e., frailty). If true, physical resilience at younger or middle ages may be predictive of future healthspan and lifespan, and provide a unique paradigm in which interventions targeting the fundamental biology of aging can be tested. This seminar will discuss research on the development of clinically-relevant measures of physical resilience in mice, including anesthesia, surgery, and cytotoxic drugs. It will further highlight how these measures compare between young, middle-aged, and older mice, and how mid-life resilience relates to later-life healthspan. Finally, it will provide insight into whether interventions targeting the biology of aging can modify physical resilience in mice. Part of a symposium sponsored by Epidemiology of Aging Interest Group.

